# Medical students’ AI literacy and attitudes towards AI: a cross-sectional two-center study using pre-validated assessment instruments

**DOI:** 10.1186/s12909-024-05400-7

**Published:** 2024-04-10

**Authors:** Matthias Carl Laupichler, Alexandra Aster, Marcel Meyerheim, Tobias Raupach, Marvin Mergen

**Affiliations:** 1 Institute of Medical Education, University Hospital Bonn, Venusberg Campus 1, 53127 Bonn, Germany; 2 Department of Pediatric Oncology and Hematology, Faculty of Medicine, Saarland University, Homburg, Germany

**Keywords:** Artificial intelligence, AI literacy, Attitudes towards AI, Confirmatory factor analysis, Medical students, Questionnaire

## Abstract

**Background:**

Artificial intelligence (AI) is becoming increasingly important in healthcare. It is therefore crucial that today’s medical students have certain basic AI skills that enable them to use AI applications successfully. These basic skills are often referred to as “AI literacy”. Previous research projects that aimed to investigate medical students’ AI literacy and attitudes towards AI have not used reliable and validated assessment instruments.

**Methods:**

We used two validated self-assessment scales to measure AI literacy (31 Likert-type items) and attitudes towards AI (5 Likert-type items) at two German medical schools. The scales were distributed to the medical students through an online questionnaire. The final sample consisted of a total of 377 medical students. We conducted a confirmatory factor analysis and calculated the internal consistency of the scales to check whether the scales were sufficiently reliable to be used in our sample. In addition, we calculated t-tests to determine group differences and Pearson’s and Kendall’s correlation coefficients to examine associations between individual variables.

**Results:**

The model fit and internal consistency of the scales were satisfactory. Within the concept of AI literacy, we found that medical students at both medical schools rated their technical understanding of AI significantly lower (*M*_*MS1*_ = 2.85 and *M*_*MS2*_ = 2.50) than their ability to critically appraise (*M*_*MS1*_ = 4.99 and *M*_*MS2*_ = 4.83) or practically use AI (*M*_*MS1*_ = 4.52 and *M*_*MS2*_ = 4.32), which reveals a discrepancy of skills. In addition, female medical students rated their overall AI literacy significantly lower than male medical students, *t*(217.96) = -3.65, *p* <.001. Students in both samples seemed to be more accepting of AI than fearful of the technology, *t*(745.42) = 11.72, *p* <.001. Furthermore, we discovered a strong positive correlation between AI literacy and positive attitudes towards AI and a weak negative correlation between AI literacy and negative attitudes. Finally, we found that prior AI education and interest in AI is positively correlated with medical students’ AI literacy.

**Conclusions:**

Courses to increase the AI literacy of medical students should focus more on technical aspects. There also appears to be a correlation between AI literacy and attitudes towards AI, which should be considered when planning AI courses.

## Background

### The rise of artificial intelligence in medicine

The potential benefits of using artificial intelligence (AI) for the healthcare sector have been discussed for decades [1–3]. However, while in the past the focus was predominantly on theoretical considerations and ambitious future scenarios, AI and its most important subfield, machine learning, have now become an integral part of healthcare [4]. In addition to clinical practice, AI applications have reached medical schools and are being used by students, educators and administrators alike to improve teaching and learning [5–6].

At the same time, a “consensus on what and how to teach AI” [7, p1] in the medical curriculum appears to be lacking, and although there are individual elective courses attempting to foster AI competencies [8–9], the majority of medical students still receive very little AI education [10–11]. However, learning basic AI skills will be critical for all future physicians to fulfill their roles as professionals, communicators, collaborators, leaders, healthcare advocates, and scholars, as all of these roles will be increasingly permeated by AI [12].

### Medical student’s “AI literacy” and related constructs

In recent years, basic AI skills have often been referred to as AI literacy [13]. AI literacy can be defined as “a set of competencies that enables individuals to critically evaluate AI technologies; communicate and collaborate effectively with AI; and use AI as a tool online, at home, and in the workplace” [13, p2]. Thus, AI literacy for medical professionals is less about the ability to develop AI programs or to conduct clinical research with AI, but rather about the ability to interact with AI and use AI applications in the day-to-day provision of healthcare services.

Despite the large number of studies investigating the attitudes and feelings of medical students towards AI (i.e., the affective component of AI interaction [14–16]),, research projects have rarely focused on AI knowledge (i.e., conceptual understanding of AI) or even AI skills (i.e., ability to identify, use, and scrutinize AI applications reasonably). Mousavi Baigi et al. [17] found that all 38 studies they included in their literature review reported some kind of investigation on healthcare students’ “attitudes towards AI” (ATAI), while only 26 of the included studies stated that they had asked participants about their AI knowledge. However, a closer look at the studies showed that most of them assessed AI knowledge superficially and focused more on familiarity with AI. Furthermore, only six of the included studies looked at the AI skills of medical students. However, since the concept of AI literacy not only encompasses AI knowledge, but also includes practical AI competencies (such as the ability to recognize the use of AI applications in technical systems), this empirical foundation is not sufficient to make reliable statements about the AI literacy of medical students.

Karaca et al. [18] were among the few who took a systematic approach to studying a closely related but not identical concept to AI literacy. They developed the so-called MAIRS-MS questionnaire instrument specifically designed to assess the “AI readiness” of medical students. AI readiness can be interpreted as a link between attitudes towards AI and knowledge and skills for dealing with AI. Aboalshamat et al. [19] used the MAIRS-MS instrument and found that medical students in a Saudi Arabian sample rated their AI readiness rather poorly with an average score of 2.5 on a Likert scale of 1 (negative) to 5 (positive). Due to the influence of socio-cultural differences and the country-specific characteristics of the medical curricula on the data, these results can only be transferred to other countries to a limited extent.

While the assessment of medical students’ AI readiness is an important endeavor, only few studies are currently dealing with competence-focused AI literacy. Evaluating these competences, however, could provide a sufficient baseline to identify knowledge gaps and, if necessary, to revise the medical curricula by developing and implementing appropriate AI courses.

### The importance of validated assessment instruments

A major disadvantage of the few available studies on the AI literacy of medical students is the attempt to assess AI literacy with self-developed and non-validated questionnaires. Thus, accuracy and reliability of their measures have not been established. In this study, we therefore used the “Scale for the assessment of non-experts’ AI literacy” (SNAIL), which was validated in several peer-reviewed studies. In a pilot study, the scale’s items were generated, refined, and subsequently evaluated for their relevance through a Delphi expert survey. As a result, a set of content-valid items covering the entire breadth of AI literacy was available to researchers and practitioners alike [20]. Subsequently, the itemset was presented to a large sample of non-experts who assessed their individual AI literacy. Based on this dataset, an exploratory factor analysis was conducted, which firstly identified the three subscales “Technical Understanding” (TU), “Critical Appraisal” (CA), and “Practical Application” (PA), and secondly excluded some redundant items [21]. In another study, it was demonstrated that the final SNAIL questionnaire is also suitable for assessing AI literacy among university students who have just completed an AI course [22].

Even though medical students’ ATAI has been assessed in multiple instances (as described above), very few studies have attempted to investigate the correlative (let alone causal) relationship between medical students’ AI literacy and ATAI. Furthermore, to our knowledge, the studies that have recorded both constructs did not use validated and standardized measurement instruments to investigate ATAI. In this study, the ATAI construct was therefore assessed using the “Attitudes towards Artificial Intelligence” scale [23], which has been validated in several languages. This scale was also developed in a systematic way, using principal component analysis and multiple samples. In addition, the reliability of the ATAI scale was evaluated and found to be acceptable. A major advantage of the scale is its efficiency, since the instrument comprises only 5 items that load on two factors (“fear” and “acceptance” of AI) in total.

### Research objective

With this study we wanted to answer five research questions (RQs). RQ1 deals with medical students’ assessment of their individual AI literacy. In particular, we aimed to assess the AI literacy sub-constructs described above (TU, CA, PA), as the identification of literacy gaps is paramount for the development of appropriate medical education programs.

RQ1: How do medical students rate their individual AI literacy overall and for the factors “Technical Understanding”, “Critical Appraisal”, and “Practical Application”?

Regarding RQ2, we wanted to investigate the extent to which the assessment of one’s own AI literacy is associated with factors such as gender, age or semester. It is conceivable, for example, that older medical students would rate their AI skills lower than younger students, as younger students might consider themselves to be more technically adept. On the contrary, older medical students might generally consider themselves to be more competent across various competence areas, as they have already acquired extensive knowledge and skills during their academic training.

RQ2: Are there statistically significant differences in AI literacy self-assessment between (a) older and younger, (b) male or female and (c) less and more advanced students?

Furthermore, the medical students’ ATAI is covered by RQ3. It is important to know whether medical students have a positive or negative attitude towards AI, as this can have a decisive influence on the acceptance of teaching programs designed to foster AI literacy.

RQ3: How do medical students rate their individual attitudes towards AI?

RQ4 follows from the ideas presented in RQ3, as it is possible that the two constructs AI literacy and ATAI are related. In addition to efforts to increase AI literacy, interventions might be required to change attitudes towards AI.

RQ4: Are the two constructs AI literacy and attitudes towards AI and their respective sub-constructs significantly correlated?

The last RQ deals with previous education and interest in AI, since both aspects might increase AI literacy. We asked if the medical students had attended courses on AI in the past or if they had already educated themselves on the topic independently. In addition, interest in the subject area of AI was surveyed.

RQ5: Is there a correlative relationship between AI education or interest in AI and the AI literacy of medical students?

## Methods

### Questionnaires

We used the “Scale for the assessment of non-experts’ AI literacy” (SNAIL) by Laupichler et al. [20] to assess the AI literacy of medical students. The SNAIL instrument assesses AI literacy on three latent factors: Technical Understanding (14 items focusing on basic machine learning methods, the difference between narrow and strong AI, the interplay between computer sensors and AI, etc.), Critical Appraisal (10 items focusing on data privacy and data security, ethical issues, risks and weaknesses, etc.), and Practical Application (7 items focusing on AI in daily life, examples of technical applications supported by AI, etc.). Each item represents a statement on one specific AI literacy aspect (e.g., “I can give examples from my daily life (personal or professional) where I might be in contact with artificial intelligence.”), which is rated on a 7-point Likert scale from 1 (“strongly disagree”) to 7 (“strongly agree”). Furthermore, we integrated the “Attitudes towards Artificial Intelligence” scale (ATAI scale) by Sindermann et al. [23]. The ATAI scale assesses participants’ “acceptance” of AI with three items and the “fear” of AI with two items. Although an eleven-point Likert scale was used in the original study, we decided to use a 7-point scale (as in SNAIL) to ensure that the items were presented as uniformly as possible. Since the sample described here consisted of German medical students, the validated German questionnaire version was used for both SNAIL [22] and ATAI [23]. All SNAIL and ATAI items were presented in random order.

We included an attention control item (“mark box 3 here.”) and a bogus item for identifying nonsensical responses (“I consider myself among the top 10 AI researchers in the world.”), which were randomly presented. Additionally, we used 4-point Likert scales to gather information on whether the students had previously taken AI courses or had educated themselves about AI through other sources. The values ranged from 1 (“I have never attended a course on AI.” and “I haven’t used other ways to learn about AI yet.”) to 4 (“I have already attended AI courses with a workload of more than 120 hours.” and “I have informed myself very extensively about AI in other ways.”). In addition, we used a 7-point Likert scale to assess students’ interest in the field of AI, with lower values indicating less interest in AI. Finally, we inquired about the participants’ age, gender, and the total number of semesters they were enrolled in their study program.

### Procedure

The study was conducted at two German medical schools (MS1 and MS2) between October and December 2023 after receiving positive ethical approval from the local ethics committees (file number 151/23-EP at medical school 1 and 244/21 at medical school 2). Invitations to participate in the study were distributed via university-exclusive social media groups and online education platforms, mailing lists, and advertisements in lectures. Medical students who were at least 18 years old were eligible for the study and could access the online questionnaire after giving their informed consent to participate. The questionnaire was accessible via a QR code on their mobile device and participants received no financial incentive to take part in the study. The average time it took respondents to complete the questionnaire was 05:52 min (SD = 02:27 min).

### Data analysis

The data were analyzed using RStudio (Posit Software, Version 2023). The visual presentation of the results was carried out using Microsoft Excel (Microsoft, Version 2016). Significance level was set at α = 0.05 for all statistical tests.

Independent two-sample t-tests were carried out to evaluate differences between groups (e.g., differences in AI literacy between MS1 and MS2). To check the requirements of t-tests, the data were examined for outliers, Shapiro-Wilk tests were carried out to check for normal distribution and Levene tests were run to check for variance homogeneity. In case of variance heterogeneity, Welch’s t-test was used. To check for differences considering age and semester distribution between MS1 and MS2, the Mann-Whitney-Wilcoxon-Test was used. Fisher’s test served to examine if there was a difference in the gender ratio.

Pearson’s correlation was calculated to determine the correlative relationship between continuous variables and Kendall’s *τ* coefficient was computed for ordinal variables. In addition, the factor structure of the two validated instruments (SNAIL and ATAI) was analyzed using a confirmatory factor analysis (CFA). We checked the prerequisites for conducting a confirmatory factor analysis, including univariate and multivariate skewness and kurtosis (using Mardia’s test for the multivariate analyses), the number and distribution of missing values, and whether the data differed significantly between the two medical schools, which would necessitate separate CFAs for each subsample. Due to the ordinal scaled variables and multivariate non-normality, we used polychoric correlation matrices to perform the CFA. We calculated the Comparative Fit Index (CFI), the Tucker-Lewis Index (TLI), the Root Mean Square Error of Approximation (RMSEA) and the Standardized Root Mean Square Residual (SRMR) as measures of model fit. As part of this analysis, the internal consistency, represented as the reliability measure Cronbach’s alpha, was also calculated for the overall scales as well as for the corresponding subscales.

## Results

### Participant characteristics

Of 444 completed questionnaires, 28 (6%) participants had to be excluded since they omitted more than 3 (10%) of the SNAIL items. In addition, 8 (2%) participants were excluded because they indicated that they did not study medicine. Furthermore, 24 (5%) participants were excluded since they did not answer or answered incorrectly to the attention control item. Finally, 7 (2%) participants had to be excluded because they agreed, at least in part, to the bogus item (i.e., counting themselves among the “Top 10 AI researchers”). Accordingly, the final sample consisted of a total of 377 (85%) subjects, of which 142 (38% of the final sample) came from MS1 and 235 (62% of the final sample) from MS2.

The participants were on average 22.5 years old (*Mdn* = 22, *Min* = 18, *Max* = 36, *SD* = 3.2) and on average in their 5th semester (*M* = 4.7, *Mdn* = 5, *Min* = 1, *Max* = 13, *SD* = 2.6). Of the participants, 259 (69%) identified as female, 114 (30%) as male and one person as diverse. A Mann-Whitney-Wilcoxon test showed that the two medical schools differed significantly from each other in terms of the age of the participants, *U* = 13658.00, *Z* = -2.63, *p* <.01. The participants in MS1 were on average 0.9 years younger than the participants in MS2. There was no significant difference regarding participants’ semesters between the two medical schools, and according to a Fisher’s test, the gender distribution was similar.

Most participants stated that they had received little or no AI training. Of all participants, 342 (91%) stated that they had never attended an AI course. Only 28 (7%) had attended a course of up to 30 h and 6 (2%) people had attended a course of more than 30 h. In addition, a total of 338 (90%) of the participants stated that they never (*n* = 177; 47%) or only irregularly (*n* = 161; 43%) educated themselves on AI using other sources (such as videos, books, etc.). Only 32 (8%) respondents stated that they regularly educated themselves on AI with the help of other sources, and only 5 (1%) participants stated that they had already educated themselves in great detail on AI.

### SNAIL and ATAI model fit

The univariate skewness and kurtosis values for the SNAIL were − 1.06 to 1.50 and − 1.08 to 1.73, which is in the acceptable range of -2.0 and + 2.0 for skewness and − 7.0 and + 7.0 for kurtosis, respectively [24]. The univariate skewness and kurtosis for the ATAI scale was also acceptable, with skewness values between − 0.45 and 0.56 and kurtosis values between − 0.68 and 0.77. Mardia’s test for multivariate skewness and kurtosis were both significant (*p* <.001), which is why multivariate non-normality had to be assumed. Due to the non-normality and the fact that the values were ordinal (because of the 7-point Likert scale), we used a polychoric correlation matrix instead of the usual Pearson correlation matrix [25]. The polychoric correlation matrix is robust against a violation of the normal distribution assumption. Since participants with a high number of missing answers were excluded before analyzing the data (see Sect. 3.1), the final data set only had an average of 1.1 missing values per variable (0.3%), which is why no data imputation was necessary.

**Fig. 1:**
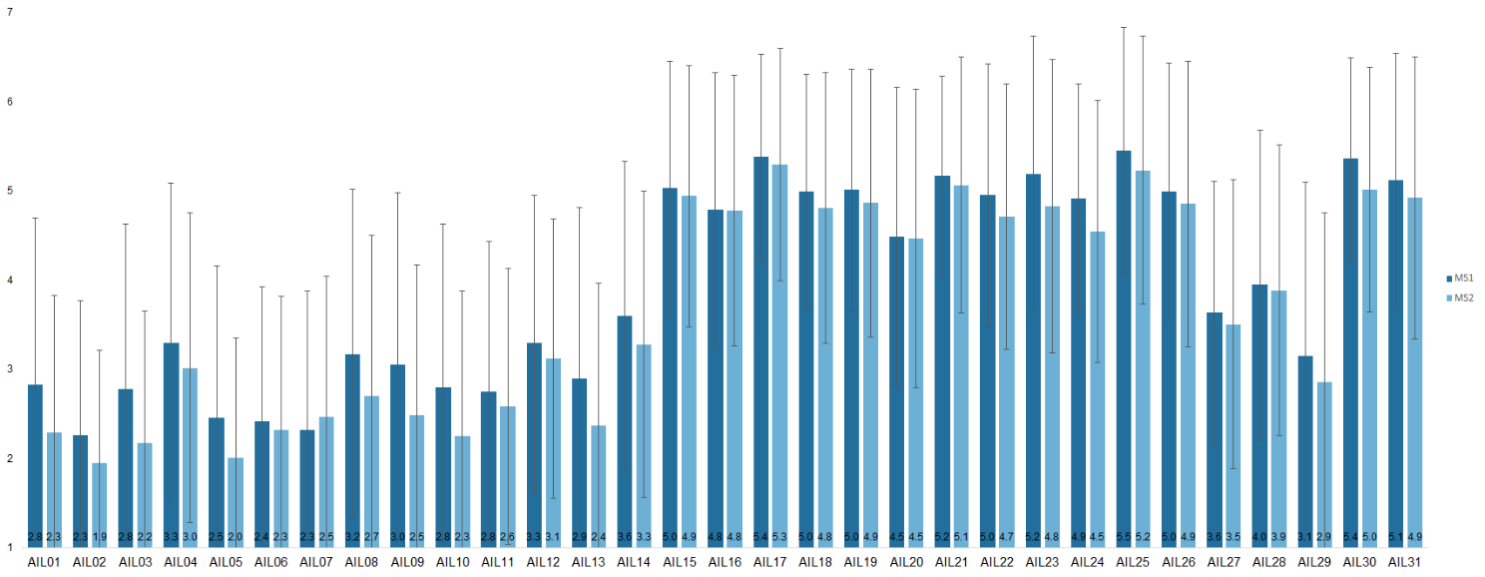
Mean score for each SNAIL item for both medical schools. *Note* Number of participants in MS1 = 142, number of participants in MS2 = 235, total *N* = 377

A t-test was performed for the SNAIL overall score, the TU, CA, and PA subscores, as well as the ATAI subscores (fear and acceptance) to check whether the data sets of the two medical schools differed significantly from each other. As the group size was much larger than *n* = 30, it could be assumed that the normal distribution assumption was not violated following the central limit theorem. A Levene test for variance homogeneity was performed for all SNAIL and ATAI scores. Since the Levene test was significant (*p* <.05) for the TU factor of the SNAIL instrument and the fear factor of the ATAI instrument, Welch’s t-test was used. Welch’s t-test showed that the overall SNAIL score, *t*(277.15) = 2.32, *p* =.02, the TU subscore, *t*(260.14) = 2.60, *p* <.01, and the fear subscore, *t*(331.36) = -2.06, *p* =.04, differed statistically significantly between the two medical schools (see Fig. 1). It was therefore decided that a separate CFA had to be carried out for the data sets of the two medical schools.

We found an equally acceptable to good model fit of the three factor model proposed by [20] for both medical schools. For MS1, the Comparative Fit Index (CFI) and Tucker-Lewis Index (TLI) were both 0.994, the Root Mean Square Error of Approximation (RMSEA) was 0.059 and the Standardized Root Mean Square Residual (SRMR) was 0.071. Accordingly, the three-factor solution fitted slightly better than a one-factor solution (i.e., a single latent factor “AI literacy”), as the latter had the following values: CFI = 0.988, TLI = 0.987, RMSEA = 0.084, SRMR = 0.083. The CFA of the MS2 data set led to comparable results. The 3-factor structure seemed to fit better with CFI = 0.994, TLI = 0.994, RMSEA = 0.059, SRMR = 0.071 than the 1-factor structure with CFI = 0.959, TLI = 0.956, RMSEA = 0.130, SRMR = 0.112. However, as expected, there is a high interfactor correlation of 0.81 between TU and CA, 0.90 between TU and PA and 0.93 between CA and PA.

Regarding ATAI, the two-factor solution proposed by Sindermann et al. [23] appears to have an excellent model fit. The following fit indices were found for MS1: CFI = 1.000, TLI = 1.012, RMSEA < 0.001, SRMR = 0.027. Excellent values were also found for MS2: CFI = 1.000, TLI = 1.016, RMSEA < 0.001, SRMR = 0.008. We found a negative interfactor correlation between “fear” and “acceptance” of − 0.83.

The internal consistency of the SNAIL subscales, expressed by the reliability measure Cronbach’s α, was good to excellent in both samples (MS1 and MS2). In the MS1 sample, the subscales had the following internal consistencies: TU, α = 0.94 [CI 0.93, 0.96]; CA, α = 0.89 [CI 0.86, 0.92], and PA, α = 0.83 [CI 0.78, 0.87]. In the MS2 sample, a Cronbach’s α of α = 0.93 [CI 0.91, 0.94] was found for the TU subscale, α = 0.89 [CI 0.87, 0.91] for the CA subscale, and α = 0.81 [CI 0.77, 0.85] for the PA subscale. However, the internal consistency of the ATAI subscales was rather low, with α = 0.53 [CI 0.35, 0.67] for the “acceptance” subscale and α = 0.61 [CI 0.48, 0.71] for the “fear” subscale in the MS1 sample and α = 0.60 [CI 0.48, 0.69] for the “acceptance” subscale and α = 0.64 [CI 0.56, 0.72] for the “fear” subscale in the MS2 sample.

### Medical students’ AI literacy (RQ1)

**Fig. 2:**
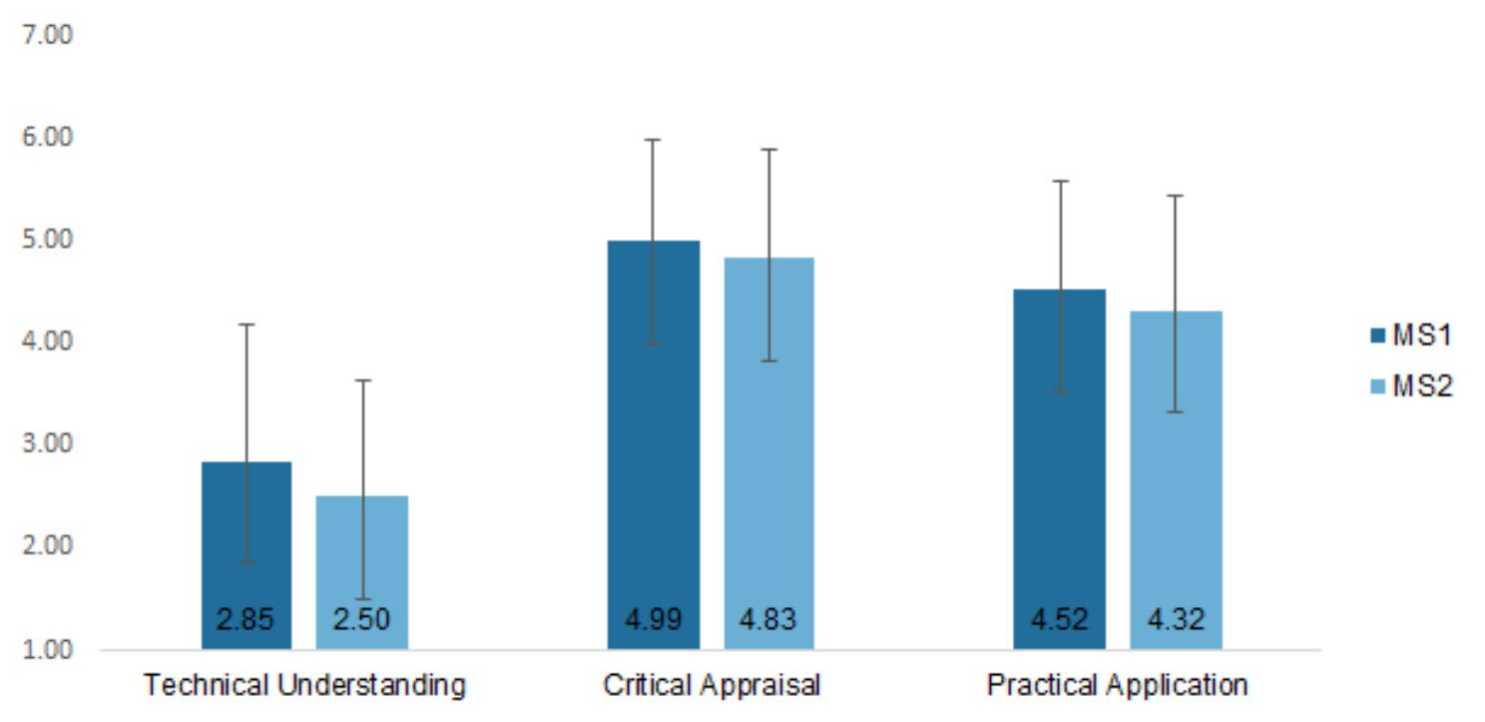
Mean score for each SNAIL factor for both medical schools. *Note* Number of participants in MS1 = 142, number of participants in MS2 = 235, total *N* = 377. MS = medical school

To determine how medical students rated their overall AI literacy, the average score of each participant was calculated for each factor as well as for the overall SNAIL scale (see Table 1). The mean TU score was 2.26 points lower than the mean CA score, *t*(734.68) = -27.26, *p* <.001, and 1.77 points lower than the mean PA score, *t*(744) = -20.86, *p* <.001. The mean CA score was 0.49 points higher than the mean PA score, *t*(750.08) = 6.28, *p* <.001. Thus, the differences between the mean values of the subscales are all statistically significant. The results of the individual analyses of the two medical schools were very similar to the overall analysis (see Fig. 2), which is why they are not reported in more detail. In the further course of this paper, the results of the individual medical schools are only given if the values differ significantly between the schools.

**Table 1:** Mean, standard deviation, skew, and kurtosis for the TU, CA, PA, and overall SNAIL score for both medical schools

		TU score	CA score	PA score	SNAIL score (all items)
MS1	*M*	2.85	4.99	4.52	3.92
	*SD*	1.33	1.00	1.07	1.08
	Skew	0.59	-0.67	-0.23	0.14
	Kurtosis	-0.49	0.85	-0.34	-0.33
MS2	*M*	2.50	4.83	4.32	3.66
	*SD*	1.33	1.07	1.11	0.99
	Skew	1.00	-0.55	-0.09	0.32
	Kurtosis	0.82	0.60	-0.18	0.27

*Note* Number of participants in MS1 = 142, number of participants in MS2 = 235, total *N* = 377. MS = medical school, TU = Technical Understanding factor, CA = Critical Appraisal factor, PA = Practical Application factor, SNAIL = Scale for the assessment of non-experts’ AI literacy

### Differences in medical students’ AI literacy due to moderator variables (RQ2)

There was no statistically significant association between the age and the average SNAIL score of participants. This applies both to the overall sample, *r* =.07, *p* =.16, as well as to the MS1 and MS2 sample, *r* =.05, *p* =.59 and *r* =.12, *p* =.07, respectively. In the overall sample, women rated their AI literacy on average 0.413 points lower than men, *t*(217.96) = -3.65, *p* <.001. There were no differences within the separate samples of the two medical schools in this respect (i.e., in both medical schools, male participants rated themselves as more AI literate). The association between the general SNAIL score and medical students’ current semester was statistically significant for the overall sample, *τ*_*c*_ = 0.08, *p* <.05. However, there was a notable difference between the two medical schools: In MS1, the association between SNAIL score and semester was not statistically significant, *τ*_*c*_ = 0.04, *p* =.52, while it was significant in MS2, *τ*_*c*_ = 0.13, *p* <.01.

### Medical students’ attitudes towards artificial intelligence (RQ3)

The participants rated their “acceptance” of AI 0.83 points higher than their “fear” of AI, *t*(745.42) = 11.72, <.001. The calculations for the MS1 and MS2 subsets led to very similar results (see Table 2).

**Table 2:** Mean, standard deviation, skew, and kurtosis for the “acceptance” and “fear” score for both medical schools

		acceptance score	fear score
MS1	Mean	4.32	3.27
	Standard deviation	0.87	0.92
	Skew	-0.48	0.07
	Kurtosis	0.09	0.05
MS2	Mean	4.19	3.49
	Standard deviation	0.96	1.07
	Skew	-0.16	0.15
	Kurtosis	0.28	-0.01

*Note* Number of participants in MS1 = 142, number of participants in MS2 = 235, total *N* = 377. MS = medical school

### Relationship between medical students’ AI literacy and attitudes towards AI (RQ4)

The SNAIL total score and the TU, CA and PA factor scores were all significantly correlated (all correlations *r* =.64 to *r* =.92, *p* <.001; see Table 3). This result indicated that the 31 items of the SNAIL questionnaire measure a common main construct, namely AI literacy.

**Table 3:** Correlation matrix for correlations between SNAIL and ATAI scores according to Kendall’s τ coefficients

	M	SD	1	2	3	4	5	6
1. SNAIL score	3.76	1.03						
2. TU score	2.63	1.22	0.92***					
3. CA score	4.89	1.05	0.90***	0.64***				
4. PA score	4.40	1.10	0.87***	0.73***	0.83***			
5. acceptance score	4.24	0.93	0.29***	0.29***	0.20***	0.28***		
6. fear score	3.41	1.02	− 0.12*	− 0.15**	− 0.03	− 0.11*	− 0.45***	

*p <.05 **p <.01 ***p <.001

*Note* All correlations shown in the table are based on the total sample (*N* = 377)

In addition, the “acceptance” subscale of the ATAI questionnaire was also significantly positively correlated with the subscales of the SNAIL questionnaire and with the total SNAIL score. The correlation between the ATAI subscale “fear” and the SNAIL scales, on the other hand, was lower and negative. “fear” correlated strongly negatively with the TU score and weakly (but still significantly) negatively with the SNAIL total score and the PA score. However, the correlation between “fear” and the CA score was not significant. Lastly, the “fear” factor of the ATAI scale correlated strongly negatively with the “acceptance” factor.

### Effect of AI education and interest on medical students’ AI literacy (RQ5)

**Fig. 3:**
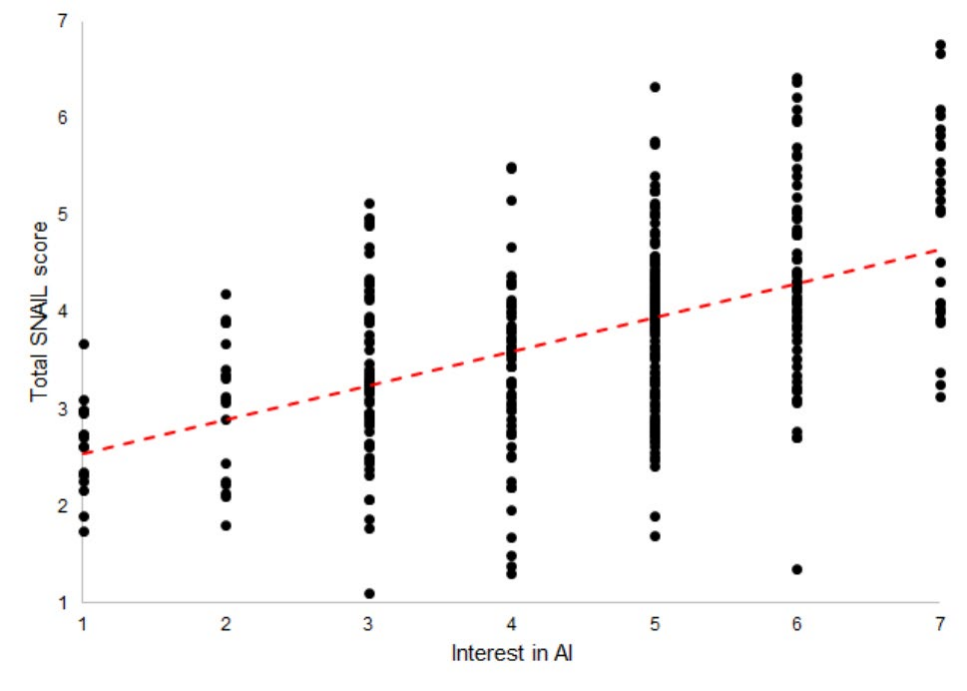
Scatterplot of Kendall’s rank correlation between the total SNAIL score and medical students’ interest in AI. *Note* The associations shown in the figure are based on the total sample (*N* = 377)

Medical students who had attended at least one shorter AI course of up to 30 h rated their AI literacy on average 1.47 points higher than medical students’ who stated that they had never attended an AI course, *t*(42.492) = 9.90, *p* <.001. The association between the two variables “Time spent attending AI courses” (ordinally scaled) and the SNAIL total score was significant, *τ*_*c*_ = 0.31, *p* <.001. In addition, students who at least irregularly used other ways to educate themselves about AI rated their AI literacy on average 0.92 points higher than students who never did so, *t*(373) = 9.70, *p* <.001. As expected, the association between the two variables “Regularity with which students train themselves on AI” (ordinally scaled) and the SNAIL total score was significant, *τ*_*c*_ = 0.43, *p* <.001. Finally, medical students’ interest in AI also appeared to be a good predictor of their AI literacy (although the causal direction of this association is not clear). Students who rated their interest in AI as rather high (5 to 7 on a 7-point Likert scale) rated their AI literacy on average 0.94 points higher than students who were less interested in AI (1 to 3 on a 7-point Likert scale), *t*(373) = 8.68, *p* <.001. The association between “Interest in AI” and the SNAIL total score was significant, *τ*_*c*_ = 0.37, *p* <.001 (see Fig. 3).

## Discussion

In this study, we assessed AI literacy and attitudes towards AI among medical students at two German medical schools using validated assessment instruments. Remarkably, medical students rated their ability to critically appraise AI and to use AI in practice as relatively high, while they rated their technical understanding of AI as rather low. In addition, although both positive and negative attitudes towards AI were evident, positive attitudes (acceptance of AI) seemed to outweigh negative attitudes (fear of AI). While the correlation between medical students’ AI literacy and acceptance of AI was clearly positive, the link between AI literacy and negative attitudes appears to be more complex.

### Interpretation and implications of the results

By using the CFA, we were able to show that the SNAIL questionnaire instrument was suitable for assessing the three latent AI literacy factors TU, CA and PA. This is evident from the good model fit of the three-factor model, but also by the excellent Cronbach’s α values for the three subscales. While the model fit was even better for the ATAI measuring instrument, Cronbach’s α of that scale was rather low, although this does not necessarily question the usefulness of the ATAI scale [26]. The low alpha values of the ATAI scale are somewhat unsurprising, considering that scales with a very small number of items also tend to have low internal consistency [27]. While the small number of items ensured good questionnaire efficiency, we could not conclusively clarify whether the five ATAI items were able to reliably assess medical students’ ATAI in our sample. Finally, we wonder whether the model fit of the ATAI model is not artificially increased, as the two subscales “acceptance” and “fear” measure practically opposite constructs. In future studies, it might therefore be advisable to recode one of the two subscales and conduct a CFA again to determine whether the two-factor structure still results in a good model fit.

RQ1 addressed the level of AI literacy and the AI literacy subconstructs TU, CA and PA of medical students. While the values of all three subscales differ statistically significantly from each other, the difference between TU and the other two factors is particularly interesting. Considering that the midpoint of a 7-point Likert scale is 4, it is surprising that the participants rated their CA and PA skills higher but their TU skills lower than the midpoint. This difference is particularly interesting because it could be assumed that a certain level of technical understanding is crucial for the practical use of AI applications. One possible explanation for the lower self-assessment score of the TU scale could be that aspects such as AI ethics, data security in connection with AI, or the recent AI hype are discussed in popular media, while technical aspects of AI, such as the function of machine learning or the difference between strong and weak AI are rather neglected.

While the age of the medical students did not appear to have any effect on their AI literacy, gender in particular had an important influence on the self-assessment of AI literacy. This is in line with a wealth of evidence suggesting that women rate themselves more negatively than men in self-assessments [28]. This effect appears to be even more pronounced for technical or scientific subjects, and negative self-assessment may even be associated with objectively lower performance [29]. Nevertheless, it is advisable to use objective AI literacy tests in addition to pure self-assessment scales in order to avoid response biases as far as possible. Furthermore, the semester also seemed to have had an influence on the self-assessment of participants’ AI literacy. The correlative relationship between the SNAIL overall score and the participants’ semester was particularly pronounced in MS2. However, a closer look reveals that in the MS2 sample, 120 participants (51% of the MS2 sample) were in semester 3 and 67 participants (29% of the MS2 sample) were in semester 7. Since 80% of the MS2 sample therefore stems from one of these two semesters, the association between semester and SNAIL score could be attributed to a sample effect.

The analyses conducted regarding RQ3 showed that medical students’ AI literacy is significantly positively correlated with their acceptance of AI, and significantly negatively correlated with their fear of AI. Thus, either AI literate medical students are more likely to accept (and less likely to fear) AI applications than AI illiterate students, or medical students who accept AI are more likely to be AI literate than students who do not accept AI. This finding complements the literature review published by Mousavi Baigi et al. [17], which found that 76% of studies reported positive attitudes towards AI among healthcare students. However, the scale midpoint of 4 should be emphasized again at this point. The medical students only “accept” AI with an average of 4.32 (MS1) and 4.12 (MS2) points and “fear” AI with 3.27 (MS1) and 3.49 (MS2) points. Although we found a statistically significant difference, it is obvious that both the negative and positive attitudes towards AI are relatively close to the midpoint. This may indicate that medical students have nuanced attitudes towards AI.

The investigation of the correlation between AI literacy and ATAI (RQ4) yielded interesting results. In the past, it has been shown for various constructs such as financial literacy [30] or scientific literacy [31] that there is a positive correlation between knowledge about a topic and positive attitudes towards it. A comparable effect was found in our study for the relationship between AI literacy and ATAI. Medical students who had a higher AI literacy were more likely to have a positive attitude towards AI (and vice versa). However, it should be mentioned again that the causality cannot be evaluated in this cross-sectional study. It is possible that medical students with a positive attitude are more willing to inform themselves about AI, resulting in a higher AI literacy. Nevertheless, it is also possible that students who are well versed in AI are better able to assess the real benefits and risks of AI, which leads to a more critical perception of exaggeratedly negative portrayals of AI.

The results regarding RQ5 indicate that courses and programs to increase AI literacy do indeed appear to have a positive effect on the AI literacy of medical students. This is an important finding as it illustrates that even relatively short AI courses (up to 30 h) are associated with higher AI literacy scores. This is particularly important in the very tightly scheduled medical curriculum, as medical AI education might be perceived as an additional burden by medical students and medical educators alike. Finally, our results indicate that the further development of curricula should arouse medical students’ interest in AI. As depicted in Fig. 3, interest in AI seems to have a strong influence on the AI literacy of medical students.

### Limitations

We have identified three main limitations: Firstly, this study was designed as a cross-sectional study which serves well to provide an initial picture of the AI literacy and ATAI of medical students. However, the correlative relationships presented here cannot provide any information about the causality of the effects. Secondly, the data was collected from two different medical schools in order to prevent sampling effects from influencing the validity of the results. Nevertheless, it is not possible to draw conclusions from the results of the two medical schools to all medical schools in Germany or even internationally, as various location factors can have an influence on AI literacy and ATAI, e.g. the current status of AI education in the medical curricula. Thirdly, all the instruments used were self-assessment questionnaires. It is conceivable that medical students’ self-assessment was subject to response biases that shifted the response behavior in one direction or the other. A bias that is particularly significant in this context is social desirability, which “refers to the tendency of research subjects to choose responses they believe are more socially desirable or acceptable rather than choosing responses that are reflective of their true thoughts or feelings” [32] (Grimm, 2010, p.1). Given that AI is a hyped topic due to recent developments such as the release of OpenAI’s ChatGPT, medical students may feel that they have at least somewhat engaged with the topic, which could potentially positively bias their response tendency. Another potential bias is the so-called acquiescence bias, which “describes the general tendency of a person to provide affirmative answers” [33]. This bias might be particularly problematic in the case of the SNAIL, as this scale has only “positive” items (i.e., higher self-assessment ratings equal higher AI literacy). However, at least the latter bias is mitigated by the fact that the SNAIL items are worded neutrally (i.e., not suggestively), which should mitigate the acquiescence tendency to some extent.

We also presented the SNAIL and ATAI items in random order and used a 7-point Likert scale for all items, as opposed to the 11-point Likert scale used by Sindermann et al. [23]. However, we believe that these adjustments to the original scales do not limit the ability of the scales to capture AI literacy and ATAI.

### Future research directions

Future studies should firstly attempt to overcome the limitations of this study and secondly continue research on AI literacy and ATAI of medical students to contribute to their better acquisition of such crucial skills.

In order to determine the causal relationships between AI literacy and ATAI or other variables (such as interest in AI), experiments should be conducted that manipulate the ATAI of medical students while establishing a control group. Longitudinal studies or randomized controlled trials would also be suitable for investigating the direction of these effects. In addition, the study should be conducted at other locations and in other countries in order to verify the generalizability of the results considering different medical curricula. Objective testing of medical students’ AI literacy [34] would also be desirable for future research projects, as objective performance measurements using knowledge or skill tests are subject to significantly less response bias. Last but not least, the development of AI education programs for medical students should be further supported and their effectiveness measured using validated scales. In this way, courses could be continuously improved to ensure that all medical students have a chance to reach a certain level of AI literacy which is required given the technological advancements. The difference between voluntary elective courses on AI and AI education as part of medical schools’ compulsory curricula would also be an important research endeavor. We call for the implementation of AI education for *all* medical students and believe that in the future all medical students should have a certain level of AI literacy in order to continue to fulfill their various professional roles in an effective and safe manner. However, this theory should be empirically tested.

## Conclusion

To our knowledge, we were the first to use validated questionnaire instruments to assess the AI literacy and ATAI of medical students. We found that medical students’ technical understanding of AI in particular was still relatively low compared to their confidence in critically evaluating and practically using AI applications. This study sheds crucial light on the AI literacy landscape among medical students, emphasizing the necessity for tailored programs. These initiatives should accentuate the technical facets of AI while accommodating students’ attitudes towards AI.

## Data Availability

The datasets used and analyzed during the current study are available from the corresponding author on reasonable request.

## Abbreviations

AI: Artificial Intelligence
ATAI: Attitudes towards AI
CA: Critical Appraisal
CFA: Confirmatory Factor Analysis
CFI: Comparative Fit Index
CI: Confidence Interval
MAIRS-MS: Medical Artificial Intelligence Readiness Scale for Medical Students
MS: Medical School
PA: Practical Application
QR: Quick-response
RMSEA: Root Mean Square Error of Approximation
RQ: Research Question
SNAIL: Scale for the assessment of non-experts’ AI literacy
SRMR: Standardized Root Mean Square Residual
TLI: Tucker-Lewis Index
TU: Technical Understanding
